# The Scientific Consensus on Climate Change as a Gateway Belief: Experimental Evidence

**DOI:** 10.1371/journal.pone.0118489

**Published:** 2015-02-25

**Authors:** Sander L. van der Linden, Anthony A. Leiserowitz, Geoffrey D. Feinberg, Edward W. Maibach

**Affiliations:** 1 Department of Psychology and Woodrow Wilson School of Public and International Affairs, Princeton University, Princeton, New Jersey, United States of America; 2 Yale Project on Climate Change Communication, School of Forestry and Environmental Studies, Yale University, New Haven, Connecticut, United States of America; 3 Center for Climate Change Communication, Department of Communication, George Mason University, Fairfax, Virginia, United States of America; University of Washington, UNITED STATES

## Abstract

There is currently widespread public misunderstanding about the degree of scientific consensus on human-caused climate change, both in the US as well as internationally. Moreover, previous research has identified important associations between public perceptions of the scientific consensus, belief in climate change and support for climate policy. This paper extends this line of research by advancing and providing experimental evidence for a “gateway belief model” (GBM). Using national data (N = 1104) from a consensus-message experiment, we find that increasing public perceptions of the scientific consensus is significantly and causally associated with an increase in the belief that climate change is happening, human-caused and a worrisome threat. In turn, changes in these key beliefs are predictive of increased support for public action. In short, we find that perceived scientific agreement is an important gateway belief, ultimately influencing public responses to climate change.

## INTRODUCTION

The scientific consensus that human activities are the primary driver of global climate change is now unequivocal. This consensus is found not only in the latest Intergovernmental Panel on Climate Change (IPCC) report [1], but also by several different studies, including surveys of experts [2] and comprehensive reviews of the peer-reviewed literature on climate change [3] [4] [5]. All of these methods converge on the same basic conclusion: at least 97% of climate scientists have concluded that human-caused climate change is happening [6].

Yet, although a scientific consensus on this basic fact has been reached, much of the public remains largely unaware of this, both in the US as well as internationally [7, 8]. For example, only one in ten Americans (12%) correctly estimate scientific agreement at 90% or higher [7]. Moreover, influential ideological and politically-motivated actors, also known as “manufacturers of doubt”, publicly dispute the existence of the scientific consensus [9, 10], including recent media articles such as the “Myth of the Climate Change 97%” [11]. These efforts to undermine public understanding of the scientific consensus have arguably been quite successful, with cascading effects on public understanding that climate change is happening, human caused, a serious threat, and in turn, support for climate change mitigation and adaptation policies.

In light of the growing ideological divide on the issue [12] (paired with people’s tendency to selectively process information), some scholars have argued that merely educating the public about the scientific consensus is unlikely to be a helpful approach [13, 14]. To better understand how people think, process and respond to the scientific consensus message, this study investigates a “gateway belief model” (GBM) of public responses to climate change.

### The Gateway Belief Model (GBM)

Perceived expert consensus plays an important role in the formation of public attitudes towards and the acceptance of general scientific principles, including climate change [15, 16]. In fact, misperceptions of the scientific consensus can be highly consequential, as even a small amount of perceived scientific dissent can undermine public support [17]. For example, a recent nationally representative study [18] found that the degree of perceived scientific agreement influences key beliefs about global warming, which in turn, drive public support for climate change policies. McCright, Dunlap & Xiao [19] successfully replicated these results in a recent independent study and similarly point to the robust role of perceived scientific agreement in generating public support for climate change policies.

**Fig 1:**
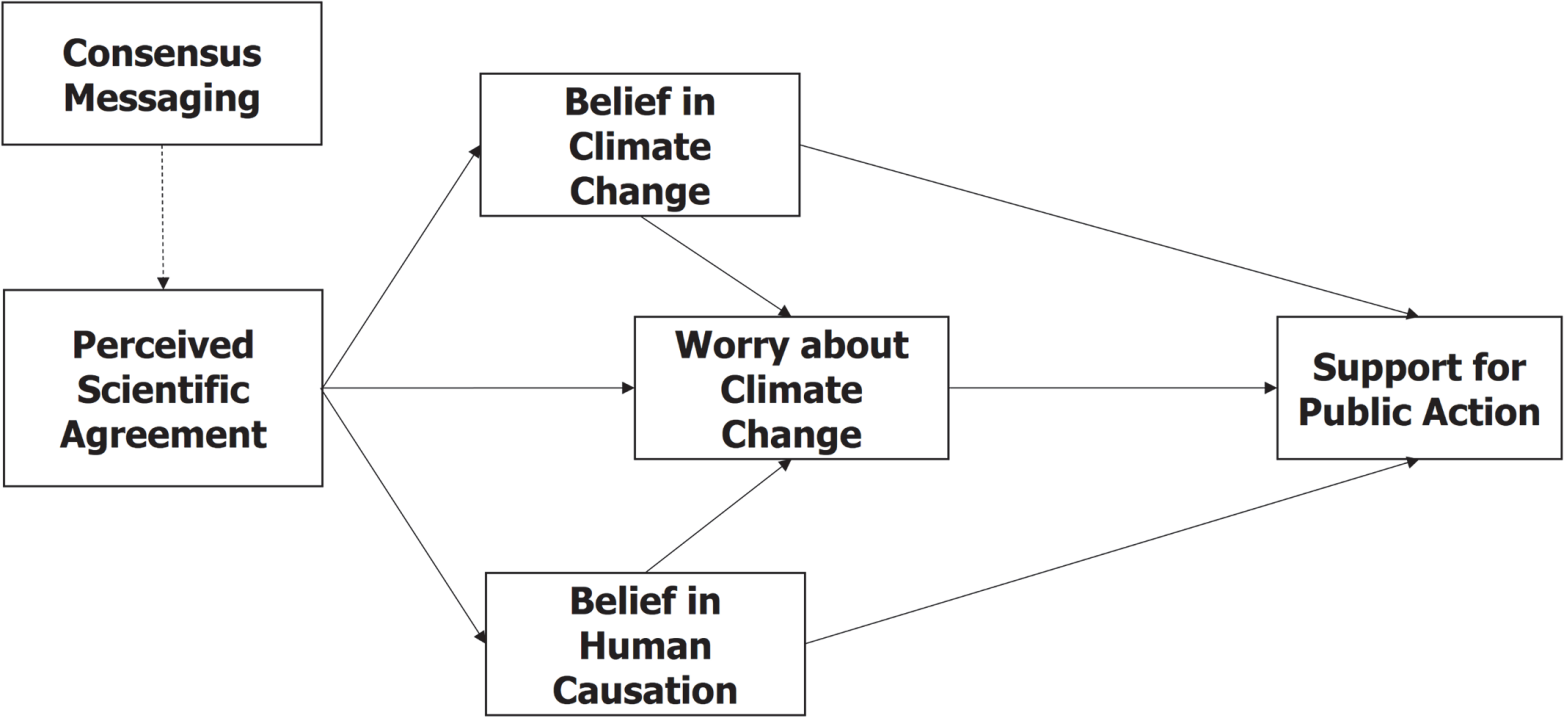
The Gateway Belief Model (GBM).

Yet, past research in this area suffers from one major short-coming: the bulk of these findings are based on cross-sectional survey data and thus correlational in nature. To date, there have been no controlled representative experiments (or longitudinal studies) investigating the proposed causal relationship between public perceptions of the scientific consensus on climate change and support for public action. This study builds upon and extends prior research in a novel direction by directly testing the “gateway belief” model experimentally. We posit that belief or disbelief in the scientific consensus on human-caused climate change plays an important role in the formation of public opinion on the issue. This is consistent with prior research, which has found that highlighting scientific consensus increases belief in human-caused climate change [15]. More specifically, we posit perceived scientific agreement as a “gateway belief” that either supports or undermines other key beliefs about climate change, which in turn, influence support for public action. A schematic overview of the “gateway belief model” is presented in Fig. 1. Specifically, we hypothesize that an experimentally induced *change* in the level of perceived consensus is causally associated with a subsequent *change* in the belief that climate change is (a) happening, (b) human-caused, and (c) how much people worry about the issue (*H1*). In turn, a change in these key beliefs is subsequently expected to lead to a *change* in respondents’ support for societal action on climate change (*H2*). Thus, while the model predicts that the perceived level of scientific agreement acts as a key psychological motivator, its effect on support for action is assumed to be fully mediated by key beliefs about climate change (*H3*).

## Method

### Sample and Participants

This analysis draws upon results from a recent experiment that investigated how to effectively communicate the scientific consensus on climate change (full details of the experiment, sample and materials are available and described in van der Linden et al. [20]). The purpose of the experiment was to test the efficacy of different ways to communicate the consensus-message (e.g., descriptive text, a pie chart, metaphors etc.). In total, 11 different treatment conditions were administered. The experiment was conducted using an online national quota sample (N = 1104) obtained from a major vendor (Survey Sampling International). The study was approved by the Yale Institutional Review Boards for ethical research (Human Research Protection Program) and participants signed a consent form with the sampling company (SSI) through which they chose to participate. A descriptive overview of the sample characteristics is provided in Table 1.

**Table 1:** Overview of sample characteristics and key belief measures.

Sample	(*N* = 1,104)		
**Demographic characteristics**			
			
Gender (female %)	52		
			
Age (modal bracket, 18, 75+)	35–44		
			
Education (bachelor's degree or higher %)	36		
			
Party Affiliation (% Democrat)	38		
			
**Key climate change beliefs (0–100)**	**Pre-Test Mean**	**Post-Test Mean**	**Difference (S.E.)**
			
Estimate of Scientific Consensus	66.98	79.72	12.74 (0.71)
			
Belief in Climate Change	73.08	77.01	3.93 (0.55)
			
Human Causation	63.98	68.02	4.04 (0.47)
			
Worry about Climate Change	62.84	67.32	4.48 (0.39)
			
Support for Public Action	75.19	76.88	1.69(0.41)

### Procedure and Materials

Subjects were asked to provide an estimate (0%–100%) of the perceived level of scientific consensus on human-caused climate change at both the beginning (pre-test) and at the end of the survey (post-test). Respondents also answered a number of questions about whether they think climate change is happening, human-caused, how worried they are about climate change and whether they think people should be doing more or less about the issue. An overview of the key belief measures used in this study is also provided in Table 1. All the consensus messages tested led to significant gains in public understanding of the scientific consensus compared to the control group. The current study, however, analyzes the data for an entirely different purpose. This study investigates whether the effect-size of the treatment messages (i.e., the *change* in respondents’ estimates of the scientific consensus) is causally associated with a pre-post *change* in the belief that climate change is happening, human-caused and a worrisome problem that requires greater societal support. To test our hypotheses, all experimental consensus-message interventions were collapsed into a single “treatment” category and subsequently compared to the “control” group. The conceptual structure of the GBM (Fig. 1) is assessed using a structural equation modeling (SEM) approach.

## Results

The path (mediation) model was estimated using STATA’s (StataCorp) SEM software. As recommended by Preacher and Hayes [21], significance of effects and model parameters was assessed using bias-corrected and accelerated bootstrap confidence intervals (the data were resampled 1,000 times). Furthermore, according to Little’s MCAR test, part of the data (approx. 8% of the sample) was missing, but not completely at random. As a result, the model was estimated using a Full Information Maximum Likelihood (FIML) procedure [22] and adjusted for important covariates, including gender, education, age and political party. Using commonly accepted criteria for model evaluation [23], the fit of the overall model structure is considered acceptable; χ^2^ (6) = 27.38, *p* < 0.01, χ^2^ / df = 4.56, CFI = 0.92, RMSEA = 0.06 (90% CI: 0.04–0.08). On average, being in one of the treatment groups (vs. the control group) significantly increases respondents’ estimate of the scientific consensus (by 12.80%). Moreover, a change in a respondent’s estimate of the scientific consensus significantly influences the belief that climate change is happening, human-caused, and the extent to which they worry about the issue (note that belief in climate change and human causation also directly influence level of “worry”). Changes in these factors, in turn, significantly predict support for public action on climate change. As hypothesized, the effect of the treatment (i.e. increased belief in the scientific consensus) on the expressed need for public action is fully mediated by the intervening variables (i.e., key beliefs about climate change). Similarly, the effect of the treatment on the key-belief measures is fully mediated by perceived scientific agreement.

**Fig 2:**
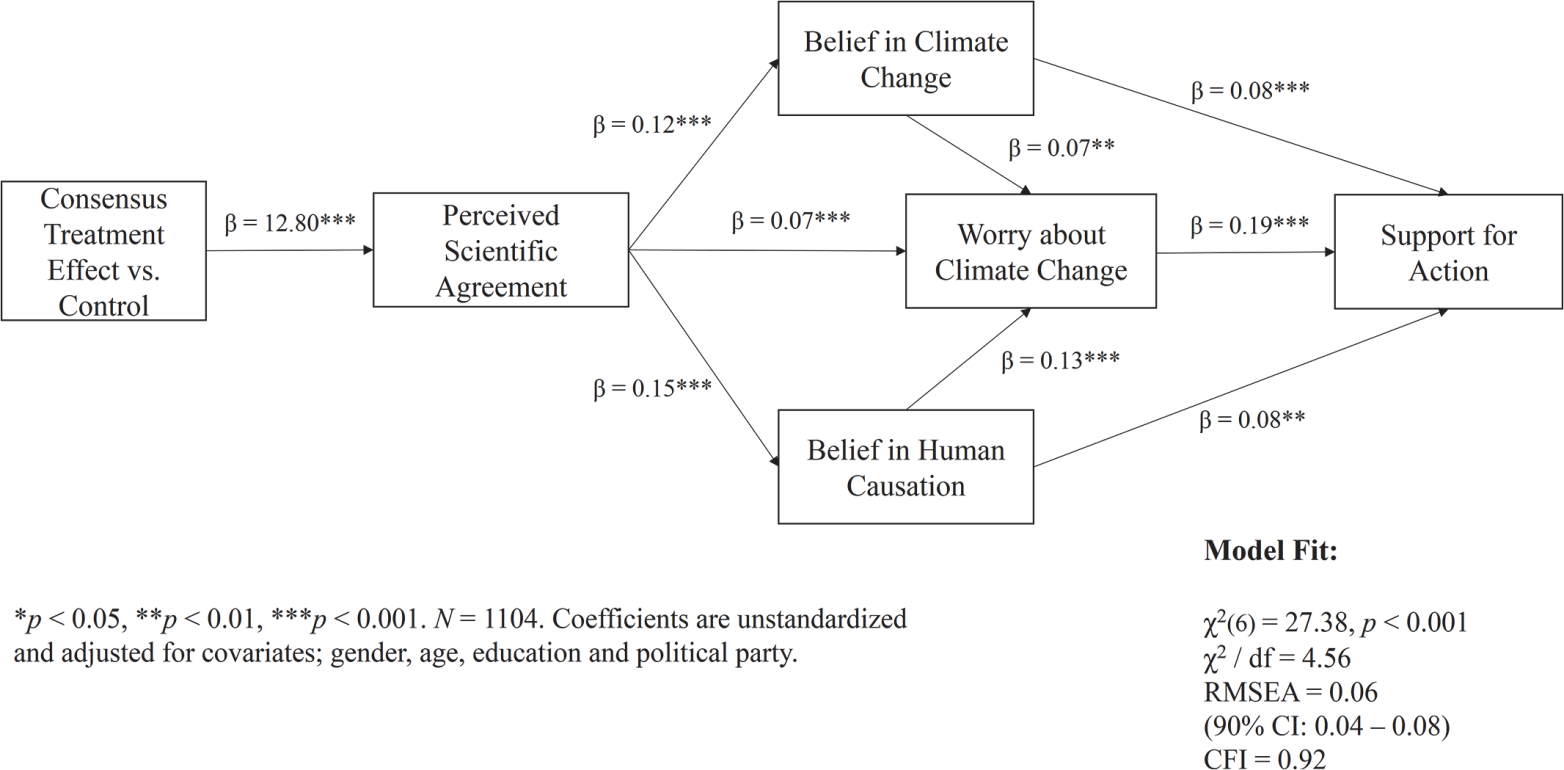
Visual depiction of the Gateway Belief Model (GBM) results.

While the model “controls” for the effect of political party, we also explicitly tested an alternative model specification that included an interaction-effect between the consensus-treatments and political party identification. Because the interaction term did not significantly improve model fit (nor change the significance of the coefficients), it was not represented in the final model (to preserve parsimony). Yet, it is important to note that the interaction itself was positive and significant (β = 3.25, SE = 0.88, *t* = 3.68, *p* < 0.001); suggesting that compared to Democrats, Republican subjects responded particularly well to the scientific consensus message. A visual depiction of the results is provided in Fig. 2 and a detailed overview of the effect sizes and model parameters is provided in Table 2.

**Table 2:** SEM model parameters.

Model path relationships	B	S.E.	95% C.I._bca_
Treatment → PSA	12.8	2.13	8.60, 17.0
			
PSA → Belief in CC	0.12	0.03	0.06, 0.16
			
PSA → Belief in HC	0.15	0.02	0.11, 0.19
			
PSA → Worry	0.07	0.02	0.03, 0.10
			
Belief in CC → Worry	0.07	0.02	0.02, 0.11
			
Belief in HC → Worry	0.13	0.03	0.07, 0.19
			
Belief in CC → Public Action	0.08	0.02	0.04, 0.12
			
Belief in HC → Public Action	0.08	0.03	0.02, 0.14
			
Worry → Public Action	0.19	0.03	0.13, 0.25
			

*Note*: Numbers are rounded. *N* = 1104. Covariates; age, gender, education and political party. PSA = Perceived Scientific Agreement; CC = Climate Change; HC = Human Causation; B; unstandardized regression coefficient, SE: standard error, 95%CI_bca_: Bias-corrected and accelerated bootstrap confidence interval (based on 1,000 bootstrap samples).

## Discussion

Previous research has suggested that perceptions of the scientific consensus play an important role in the formation of public beliefs and attitudes towards climate change and, moreover, that (mis)perceptions of the scientific consensus potentially decrease public support for climate change policies [15–19]. This study constructively builds upon and extends this research by providing direct experimental evidence for the “gateway belief model” (GBM). Using pre and post measures from a national message test experiment, we found that all stated hypotheses were confirmed; *increasing* public perceptions of the scientific consensus causes a significant increase in the belief that climate change is (a) happening, (b) human-caused and (c) a worrisome problem. In turn, changes in these key beliefs lead to increased support for public action. In sum, these findings provide the strongest evidence to date that public understanding of the scientific consensus is consequential.

It is important to note that the gateway belief model (GBM) describes a two-step cascading effect. First, the effect of consensus messaging on key beliefs about climate change is fully mediated by the perceived level of scientific agreement. Second, the effect of the induced increase in perceived scientific consensus is fully mediated onto support for public action via the key beliefs about climate change. In other words, belief in the scientific consensus functions as an initial “gateway” to changes in key beliefs about climate change, which in turn, influence support for public action. Thus, consistent with other recent research, this study found that when in doubt about scientific facts, people are likely to use consensus among domain experts as a heuristic to guide their beliefs and behavior [15].

These findings have important practical implications for science communication and stand in direct juxtaposition to the claim that “consensus-messaging” is not effective as a communication strategy [13, 14]. In particular, it is sometimes argued that (a) despite past public communication efforts, public understanding of the scientific consensus has not changed much in the last decade and hence the approach must not be very effective (i.e., “the stasis argument”) [13] and (b) because people are predisposed to engage in protective motivated reasoning (i.e., people process information consistent with their ideological worldviews), consensus-messaging is likely to be unsuccessful or could even backfire [12, 14].

The present study finds no support for these claims. On the contrary, results of this study show that perceived scientific consensus acts as a key gateway belief for both Democrats and Republicans. In fact, the consensus message had a larger influence on Republican respondents. It should be noted that this interaction might, to some extent, be attributable to a ceiling effect (i.e., there is relatively less upward adjustment potential in perceived scientific consensus for Democrats, although a significant gap in understanding persists even among Democrats). We do not dispute, however, that some people—especially those with strong ideological responses to the issue—selectively process information or engage in motivated reasoning [9, 14]. Yet, we find that consensus-messaging does not increase political polarization on the issue (perhaps partly due to the neutral scientific character of the message) and shifts the opinions of both Democrats and Republicans in directions consistent with the conclusions of climate science.

Furthermore, other recent research [24] has suggested that past campaigns have been unsuccessful (in both their reach and exposure), given that a substantial lack of awareness of the scientific consensus still persists (“information deficit”) while at the same time, the spread of misinformation has vastly increased (“misinformation surplus”). Because people often encounter multiple and conflicting informational cues, the criticism might be raised that as a controlled experiment, this study may overstate the actual effect that consensus messaging would have in the real-world. While we agree with this view and see this as an important and open area for future research, this shortcoming does not, however, negate the structural validity of the GBM’s causal mechanisms. It is also important to note that this study only used a single treatment, yet found that even a single, simple description of the scientific consensus significantly shifted public perceptions of the consensus and subsequent climate change beliefs and desire for action. A concerted campaign to inform the public about the scientific consensus would ideally involve numerous exposures to the key message, conveyed by a variety of trusted messengers [6, 20].

This is important because by strategically sowing seeds of doubt, organized opponents of climate change action have continually tried to undermine the validity of the scientific consensus argument [11]. As this research shows, such attempts could potentially decrease public engagement with climate change. Nonetheless, the present research also indicates the potential efficacy of consensus-messaging campaigns in mitigating such skepticism, as well as in generating support for public action on climate change. Particularly, repeated exposure to simple messages that correctly state the actual scientific consensus on human-caused climate change is a strategy likely to help counter the concerted efforts to misinform the public. Effectively communicating the scientific consensus can also help move the issue of climate change forward on the public policy agenda [6] [15] [20] [24–25].

## Supporting Information

S1 File (PDF)
