# Supplementary material for: The Scientific Consensus on Climate Change as a Gateway Belief: Experimental Evidence
Source: PLoS One. 2015 Feb 25;10(2):e0118489. doi: 10.1371/journal.pone.0118489 (PMC4340922; doi:10.1371/journal.pone.0118489)
Supplement: S1 File — (PDF) [file pone.0118489.s001.pdf]

Climatic Change (2014) 126:255–262
DOI 10.1007/s10584-014-1190-4

LETTER

How to communicate the scientific consensus on climate change: plain facts, pie charts or metaphors?

Sander L. van der Linden · Anthony A. Leiserowitz · Geoffrey D. Feinberg · Edward W. Maibach

Received: 19 April 2014 / Accepted: 30 June 2014 / Published online: 6 July 2014
© Springer Science+Business Media Dordrecht 2014

Abstract Previous research has identified public perceptions of the scientific consensus on climate change as an important gateway belief. Yet, little research to date has examined how to effectively communicate the scientific consensus on climate change. In this study, we conducted an online experiment using a national quota sample to compare three approaches to communicating the scientific consensus, namely: (a) descriptive text, (b) a pie chart and (c) metaphorical representations. Results indicate that while all three approaches can significantly increase public understanding of the degree of scientific consensus, the pie chart and simple text have superior recall and are most effective across political party lines. We conclude that the scientific consensus on climate change is most effectively communicated as a short, simple message that is easy to comprehend and remember. Representing the consensus visually in the form of a pie chart appears to be particularly useful.

1 Introduction and context

The latest Intergovernmental Panel on Climate Change (IPCC) report states with 95 % certainty that human-caused climate change is happening (IPCC 2013). The IPCC is not alone in its conclusion: a recent review of over 12,500 scientific peer-reviewed abstracts on the subject of global climate change found that 97 % of the papers accept the consensus position that humans are causing global warming (Cook et al. 2013) – a finding consistent with results of earlier surveys of the scientific literature on climate change (Oreskes 2004; Anderegg et al. 2010; Doran and Zimmerman 2009).

Electronic supplementary material The online version of this article (doi:10.1007/s10584-014-1190-4) contains supplementary material, which is available to authorized users.

S. L. van der Linden (✉) · A. A. Leiserowitz · G. D. Feinberg
Yale Project on Climate Change Communication. School of Forestry and Environmental Studies, Yale University, 195 Prospect Street, New Haven, CT 06511, USA
e-mail: sander.vanderlinden@yale.edu

E. W. Maibach
Center for Climate Change Communication and Department of Communication, George Mason University, 4400 University Dr, New Fairfax, VA 22030, USA
e-mail: emaibach@gmu.edu

While the consensus among climate scientists is now nearly unanimous, the American public remains largely unaware of this fact. Indeed, only 42% of Americans believe that "most climate scientists think global warming is happening" and only 22 % correctly estimate the scientific consensus (in quintiles) between 81 and 100 % (Leiserowitz et al. 2014). Similarly, an international survey across 16 nations indicated that only 51 % of respondents believed that "most scientists think the problem is urgent and enough is known to take action" (World Public Opinion Poll 2009).

The importance of correcting such widespread misunderstandings about the scientific consensus is currently an active area of scholarly debate. For example, the assumption that a lack of public engagement with climate change is due to a gap in knowledge or public understanding (the so-called "knowledge-deficit model") has been questioned (Moser and Dilling 2011; Sturgis and Allum 2004). Indeed, Kahan et al. (2012) argue that divisions over climate change have little to do with the public's comprehension of the scientific evidence. Yet, other recent research has repeatedly shown that the perceived level of scientific agreement on human-caused climate change actually functions as a critical "gateway belief" (Ding et al. 2011; Lewandowsky et al. 2013; McCright et al. 2013). Importantly, the scientific consensus is a different and very specific type of public knowledge – knowledge of the level of agreement among domain experts that the phenomenon exists – not direct knowledge about climate change itself (e.g., the role of carbon dioxide, parts per million in the atmosphere etc.). Moreover, (mis)perceptions of the scientific consensus are highly consequential, as they often lead to incorrect beliefs about the reality and causes of climate change among many members of the public, and in turn, (can) diminish support for climate change policies (Aklin and Urpelainen 2014; Ding et al. 2011; McCright et al. 2013). While some scholars still maintain that people are unlikely to change their prior held beliefs when presented with simple information about the scientific consensus (e.g. Kahan et al. 2011), this study was explicitly designed to test this assumption.

There are a number of potential strategies to communicate the scientific consensus on climate change. Perhaps the easiest and most basic strategy is to simply state the literal scientific facts in descriptive text (i.e., "97 % of climate scientists have concluded that human-caused climate change is happening"). Although the available experimental evidence is very limited, there at least appears to be some marginal support for this approach (Bolsen et al. 2013).

A more popular technique, however, is to represent information about the consensus in the form of a classic pie chart, as is currently done by the "Consensus Project" (Consensus Project, 2013). While very little research has examined how effective this device actually is in communicating the climate science consensus (Lewandowsky et al. 2013), there is strong evidence for the utility of graphics as a means to present quantitative information more generally. For example, visual graphics can attract and hold people's attention, help the observer process information more effectively and facilitate memory function (Lipkus and Hollands 1999; Tverksy 2001). In fact, compared to other types of graphics, several psychophysical studies have suggested that proportions are most effectively processed when presented in the form of a pie chart (Hollands and Spence 1999; Spence and Lewandowsky 1999).

A third alternative is to harness the power of metaphor. A metaphor is an implied comparison between two dissimilar objects that lets people experience one thing in terms of another. Metaphors are well-known to enhance persuasion (Sopory and Price-Dillard 2002), and, through their communicative and connective function, they can help generate new insights and make complex concepts more meaningful (Lakoff 1993; Lakoff and Johnson 1980; Mio 1997). Because of their ability to make abstract concepts more relatable, the use of metaphor is ubiquitous in climate change discourse: there are greenhouse effects, atmospheric blankets, time bombs, tipping points, and overflowing bath tubs, just to name a few (Russill 2011). However, thus far, the use of metaphor has not been explored as a means to represent

the scientific consensus on climate change (e.g. by drawing comparisons to more commonly accepted forms of expert agreement).

Accordingly, the present research addresses an important gap in the literature by directly contrasting the effectiveness of each of these three approaches in a controlled experimental setting. More specifically, we set out to investigate two research questions. First, to what extent can descriptive text, pie charts and metaphors influence people's estimate of the scientific consensus? Second, do any of these three approaches have a comparative advantage in terms of their ability to improve public understanding?

2 Method

To answer these questions, we conducted a randomized online survey experiment in July of 2013 using a national quota sample ($N=1104$) based on gender and age. Participants were recruited through Survey Sampling International, a vendor that maintains a nationwide panel of people who are willing to participate in online surveys. The experiment included a (pre/post) between-subject design with a total of eleven treatment conditions, each of which contained approximately 100 participants. In order to disguise the real purpose of the experiment, subjects were informed at the start of the survey that they were participating in a public opinion poll about popular topics. Participants were subsequently given a practice question to make sure they understood the answer format.

In order to make the survey look like a public opinion poll, two bogus question blocks about contemporaneous news stories were included (of equal length). The first block asked subjects for their opinion about Angelina Jolie's double mastectomy, the second block surveyed their opinion on the National Transportation Safety Board's recommendation to reduce the "drunk-driving" blood-alcohol level, while the third block asked participants about their perceptions and beliefs about climate change. The two bogus topics were chosen for their relatively neutral political character and recent coverage in the media. All blocks were presented in random order.

In addition, all subjects were presented with the exact same question set before (pre) and after (post) the treatments were administered. After the pre-test, participants were further "distracted" with an unrelated article about the upcoming 2014 Star Wars Animated Series. Following the article, subjects were told that they would be randomly shown one of several messages (at this point, participants were randomly allocated to one of the eleven treatment groups). All treatments contained the following message; "*97% of climate scientists have concluded that human-caused climate change is happening*". To enhance the credibility of the treatment, the logo of the American Association for the Advancement of Science (AAAS) was visible on every message.

In order to test the effectiveness of metaphors as a communication device, we used a total of eight metaphor conditions (Fig. 1). The metaphors were designed using a "risk management" frame (NRC 2011) and in doing so, we followed the guidelines of a relevant meta-review which (for optimal persuasiveness) recommends the use of single (non-extended), novel metaphors with a familiar target (Sopory and Price-Dillard 2002). Two variations were used to establish an implied comparison with an easily relatable and common type of expert consensus, namely; a "doctor metaphor" (i.e., "*If 97% of doctors concluded that your child is sick, would you believe them? 97% of climate scientists have concluded that human-caused climate change is happening*") and a "bridge metaphor" (i.e., "*If 97% of engineers concluded that a particular bridge is unsafe to cross, would you believe them? 97% of climate scientists have concluded that human-caused climate change is happening*"). The metaphors were further subdivided into *text-only* vs. *full* (text + image) and *belief* vs. *action* metaphors (e.g., "*If 97% of doctors concluded that your child was sick, would you have your child treated?*").

Fig. 1 Overview of metaphor treatment conditions

	Doctor		Bridge	
	Full Metaphor	Text-Only	Full Metaphor	Text-Only
**Belief**	I	II	III	IV
**Action**	V	VI	VII	VIII

The remaining treatments consisted of just the descriptive text describing the degree of consensus and a pie chart representing the consensus visually. Participants in the control group received no information. Examples of the treatments are provided as supplementary information.

The main dependent variable was a subject's (pre and post) estimate of the current level of scientific consensus on human-caused climate change (0–100 %). In order to avoid common method bias (Podsakoff et al. 2003), psychological separation of measurement was achieved by maximizing the distance between the pre and post-test measures. In addition, using the same scale (0–100), subjects were also asked how certain they were about their estimate, how likely they think it is that climate change is happening, to what extent they believe it is human caused, whether they worry about it and whether people should be doing more or less about climate change. Lastly, a brief manipulation check was administered to assess how well the participants recalled the information that was presented to them. An overview of the sample characteristics and key climate change belief measures is provided as part of the supplementary information.

3 Results

Within-subject differences in estimates of the level of scientific consensus are reported in Table 1 (by treatment) and range from +9.51 % (minimum) to +17.88 % (maximum). On average, across all conditions, the treatments increased subjects' estimates of the scientific consensus by 12.74 % (compared to 0.04 % in the control group). To test whether the differences between the groups are significant, we conducted an analysis of covariance (ANCOVA) with the post-test as the dependent variable and the pre-test as the covariate. Note that the post-test in this case is statistically equivalent to the difference score (Bonate 2000).

The ANCOVA revealed a significant main effect for the treatment levels, $F(10, 1076)=6.75$, MSE=350.76, $p<0.01$, $\eta_p^2 = 0.06$. Post-hoc comparisons on the adjusted marginal means using the Tukey HSD test indicated positive and significant differences for all 11 treatments when compared to the control group ($p<0.01$). None of the metaphor conditions differed significantly from each other.

To further examine differences between the metaphor treatments, the eight metaphors were recoded into two equal groups; text-only metaphors ($\bar{x}$ =11.85) and full (text + image)

Table 1 Overview of pre-post differences in estimates of the scientific consensus by treatment group

Treatment conditions	Estimate of scientific consensus % (pre-test mean)	Estimate of scientific consensus % (post-test mean)	Difference (post test – pre test)%	S.E. (diff)
Control group (n=115)	66.97	66.93	0.04	1.42
Descriptive text (n=87)	65.94	83.82	17.88	2.64
Pie chart (n=102)	70.71	85.09	14.38	2.11
Action metaphor – doctor full (n=98)	62.98	77.35	14.37	2.23
Belief metaphor – doctor text-only (n=120)	65.32	79.62	14.30	2.27
Belief metaphor – bridge full (n=87)	68.46	81.39	12.93	2.54
Action metaphor – doctor text-only (n=88)	68.02	79.50	11.48	2.27
Action metaphor – bridge text-only (n=86)	65.40	76.52	11.12	2.30
Belief metaphor – bridge text-only (n=106)	71.02	81.87	10.85	2.03
Action metaphor – bridge full (n=99)	66.41	77.01	10.60	1.92
Belief metaphor – doctor full (n=116)	66.16	75.67	9.51	2.16
Overall average	66.98	79.72	12.74	0.71

metaphors ($\bar{x}$ = 11.94). No meaningful differences emerged (the same holds true for other categorization schemes, e.g., doctor vs. bridge or belief vs. action). Given the relative homogeneity of the metaphor-effect and in order to reduce the number of multiple comparisons, all metaphors were collapsed into one condition (Table 2) and compared to the remaining treatment groups (the pie chart, descriptive text and the control group).

Similarly, the ANCOVA revealed a significant main effect for the reduced set of comparisons, $F(3, 1083)=20.51$, MSE=350.43, $p<0.01$, $\eta_p^2 = 0.05$. We used the Tukey-Kramer adjustment for unbalanced groups to perform post hoc comparisons on the adjusted marginal means. Results indicate that compared to the metaphor group, both the pie chart and the descriptive text conditions elicited significantly higher estimates of the scientific consensus ($p<0.05$). The difference between the pie chart and the descriptive text was not significant.

Table 2 Overview of pre-post differences in estimates of the scientific consensus by (recoded) treatment conditions

Treatment conditions	Estimate of scientific consensus (pre-test mean) %	Estimate of scientific consensus (post-test mean) %	Difference test – pre test) %	S.E. (diff)
Control group (n=115)	66.97	66.93	0.04	1.42
Descriptive text (n=87)	65.94	83.82	17.88	2.64
Pie chart (n=102)	70.71	85.09	14.38	2.11
Average of metaphors (n=800)	66.72	78.62	11.91	0.79
Overall average	66.98	79.72	12.74	0.71

We also administered a manipulation check to assess to what extent the participants correctly remembered the information that was presented to them. Results indicated a significant difference between the conditions, $F(1, 974)=7{,}54$, $p<0.01$. On average, the recall of subjects who were in the descriptive text or pie chart conditions was modest but significantly closer to the 97 % consensus figure ($\bar{x}= 87.67$, SE=1.22) than of those who were in one of the metaphor conditions ($\bar{x}= 83.17$, SE=0.74).

Additionally, a significant interaction emerged between treatment group and political affiliation $F(6, 1346)=3.98$, $p<0.01$, $\eta_p^2 = 0.03$. Results are presented below in Fig. 2. Consistent with prior results, all groups across political party lines responded most positively to the pie chart and the descriptive text. However, while Republicans, Democrats and Independents all responded relatively similarly to the metaphors, Republicans tended to respond more strongly to the pie chart (compared to both Democrats and Independents).

A main effect was also found for *certainty of the estimate*, $F(10, 1027)=5.97$, MSE=569.31, $p<0.01$, $\eta_p^2 = 0.05$. Post-hoc comparisons on the adjusted marginal means using the Tukey HSD test indicated that all 11 treatments increased certainty of belief-estimates when compared to the control group ($p<0.01$). For the reduced set of comparisons, the relative gains in belief-certainty were as follows; 14 % (*average of metaphors*), 18 % (*descriptive text*) and 18 % (*pie chart*), respectively. Differences between treatment groups were not significant.

Lastly, pre-post differences between the treatment groups for the remaining constructs (i.e. belief in climate change, the extent to which it is human-caused, worry and need for action) were also examined, but found to be statistically non-significant at conventional levels and therefore not further reported here.

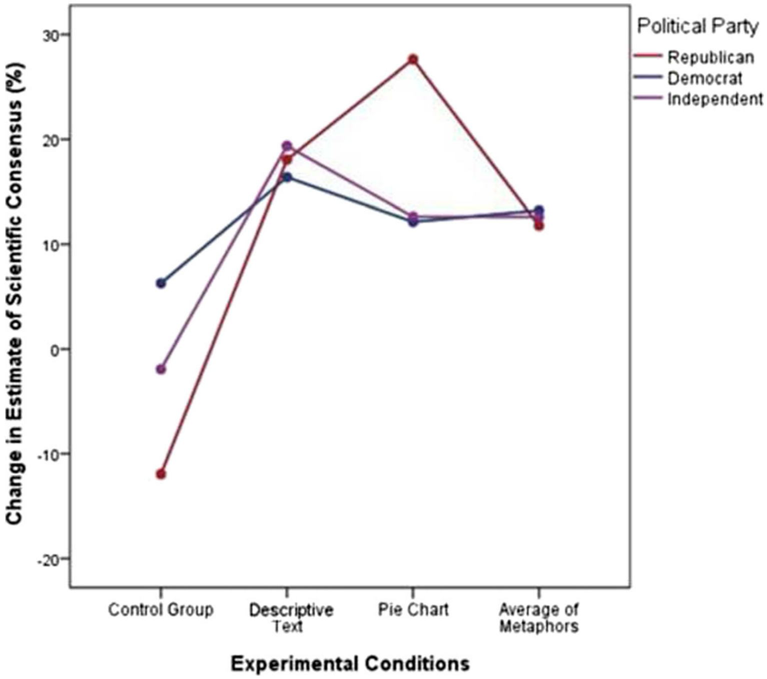

Fig. 2 Overview of mean (pre-post) differences in estimates of the scientific consensus by political party

4 Discussion

Results of this study lead to two major conclusions. First, regardless of whether the message is in the form of a metaphor, descriptive text or pie chart, providing people with information about the scientific consensus on human-caused climate change is effective in and of itself and can significantly increase people's estimates of the scientific consensus (as well as belief-certainty associated with those estimates). Second, as a communication device, the pie chart and descriptive text were most effective at conveying the scientific consensus on climate change. Compared to the metaphor conditions, participants in the pie chart and descriptive text conditions adjusted their estimates upward at a significantly higher rate (this effect was particularly strong for Republican participants). The manipulation check clearly suggests one explanation for these findings; the descriptive text and pie chart both generated better recall of the content of the scientific consensus message (i.e., 97 %) - which is congruent with earlier discussed research on visual processing and memory function (Lipkus and Hollands 1999; Tverksy; 2001; Spence and Lewandowsky 1999; Hollands and Spence 1999).

Taken as a whole, these results are somewhat surprising in light of the frequent use of metaphors in climate change discourse. A plausible explanation for their relatively modest influence is that the use of metaphor might be especially useful in explaining complex (causal) mechanisms such as the phenomena of stock and flow and accumulation (Guy et al. 2013), whereas the use of metaphor may be less effective when explaining relatively simple and straightforward concepts such as the degree of scientific consensus on climate change. Nonetheless, compared to the control group, all of the metaphor conditions led to higher estimates of the scientific consensus.

In conclusion, the results of this study indicate that simple objective information about the scientific consensus can effectively change people's prior held beliefs and perceptions about the consensus. In particular, the results suggest that when communicating the scientific consensus on human-caused climate change, presenting information in a way that is short, simple and easy to comprehend and remember seems to offer the highest probability of success for all audiences examined. To this extent, the conventional pie chart functions as a particularly effective and visually appealing communication device. In light of prior research that has identified perceptions of the scientific consensus as an important gateway belief (Ding et al. 2011; McCright et al. 2013; Lewandowsky et al. 2013; Aklin and Urpelainen 2014), the current study provides practical guidelines on how to communicate this fact.

One potential limitation of this study is its "ecological validity". For example, although the present study did investigate how different political audiences responded to the treatment messages, people often simultaneously process multiple and conflicting informational cues from different sources. Moreover, beliefs, opinions and perceptions change and evolve dynamically over time. Future research could therefore assess the robustness of changes in perceptions of the scientific consensus longitudinally, as well as explore how the proposed communication strategies function outside of the laboratory in more "politically contested" environments.

Acknowledgments This research was funded by the American Association for the Advancement of Science (AAAS) and the Rockefeller Family Fund (RFF) as well as by Lawrence Linden, Robert Litterman and Henry Paulson.

Author contributions All authors contributed to the conceptualization of the study and research questions. S.L.V., A.A.L., and G.D.F. designed and conducted the survey. S.L.V performed the data analysis and wrote the first draft of the manuscript. A.A.L., G.D.F., and E.W.M wrote, commented on and revised parts of the manuscript.

Competing financial interests The authors declare no competing financial interests.

References

Aklin M, Urpelainen J (2014) Perceptions of scientific dissent undermine public support for environmental policy. Environ Sci & Policy 38:173–177

Anderegg WRL, Prall JW, Harold J, Schneider SH (2010) Expert credibility in climate change. Proc Natl Acad Sci U S A 107:12107–1209

Bolsen T, Leeper TJ, Shapiro MA (2013) Doing what others Do: norms, science, and collective action on global warming. Am Polit Res 42:65–89

Bonate P (2000) Analysis of Pretest-Posttest Designs. Chapman & Hall/CRC.

Consensus Project (2013) http://theconsensusproject.com/. Accessed 10 January 2014

Cook J et al (2013) Quantifying the consensus on anthropogenic global warming in the scientific literature. Environ Res Lett 8:024024

Ding D, Maibach EW, Zhao X, Roser-Renouf C, Leiserowitz A (2011) Support for climate policy and societal action are linked to perceptions about scientific agreement. Nature Clim Chang 1:462–465

Doran PT, Zimmerman MK (2009) Examining the scientific consensus on climate change. EOS Trans Am Geophys Union 90:21–22

Guy S, Kashima Y, Walker I, O'Neill S (2013) Comparing the atmosphere to a bathtub: effectiveness of analogy for reasoning about accumulation. Clim Chang 121:579–594

Hollands JG, Spence I (1999) Judging proportions with graphs: the summation model. Appl Cogn Psychol 12: 173–190

IPCC (2013) Climate change 2013: the physical science basis. Contribution of working group I to the fifth assessment report of the intergovernmental panel on climate change. Cambridge University Press, Cambridge

Kahan DM, Jenkins-Smith H, Braman D (2011) Cultural cognition of scientific consensus. J Risk Res 14:147–174

Kahan DM, Peters E, Wittlin M, Slovic P, Ouellette LL, Braman D, Mandel G (2012) The polarizing impact of science literacy and numeracy on perceived climate change risks. Nature Clim Chang 2:732–735

Lakoff G (1993) The contemporary theory of metaphor. In: Ortony A (ed) Metaphor and thought, 2nd edn. Cambridge University Press, Cambridge, pp 203–251

Lakoff G, Johnson M (1980) Metaphors We live by. University of Chicago Press, Chicago

Leiserowitz A, Maibach E, Roser-Renouf C, Feinberg G, Rosenthal S, Marlon J (2014) Climate Change in the American Mind: American's Global Warming Beliefs and Attitudes in November 2013. Yale Project on Climate Change Communication, New Haven, CT. http://environment.yale.edu/climate-communication/files/Climate-Beliefs-November-2013.pdf

Lewandowsky S, Gignac GE, Vaughan S (2013) The pivotal role of perceived scientific consensus in acceptance of science. Nature Clim Chang 3:399–404

Lipkus IM, Hollands JG (1999) The visual communication of risk. J Natl Cancer Inst Monogr 25:149–163

McCright AM, Dunlap RE, Chenyang X (2013) Perceived scientific agreement and support for government action on climate change in the USA. Clim Chang 119:511–518

Mio J (1997) Metaphor and politics. Met Symb 12:113–133

Moser SC, Dilling L (2011) Communicating climate change: closing the science-action gap. In: Dryzek JS, Norgaard RB, Schlosberg D (eds) The oxford handbook of climate change and society. Oxford University Press, Oxford, pp 161–174

National Research Council (2011) America's climate choices. The National Academies Press, Washington, DC

Oreskes N (2004) Beyond the ivory tower: the scientific consensus on climate change. Science 306:1686

Podsakoff PM, MacKenzie SB, Lee Y-J, Podsakoff NP (2003) Common method biases in behavioral research: a critical review of the literature and recommended remedies. J Appl Psychol 8:879–903

Russill C (2011) Temporal metaphor in abrupt climate change communication: an initial effort at clarification. In: Filho WL (ed) The economic, social and political elements of climate change. Climate Change Management. Springer, Heidelberg, pp 113–132

Sopory P, Price-Dillard J (2002) The persuasive effects of metaphor: a meta-analysis. Human Commun Res 28: 382–419

Spence I, Lewandowsky S (1999) Displaying proportions and percentages. Appl Cogn Psychol 5:61–77

Sturgis P, Allum N (2004) Science in society: re-evaluating the deficit model of public attitudes. Public Underst Sci 13:55–74

Tverksy B (2001) Spatial schemas in depiction. In: Gattis M (ed) Spatial schemas and abstract thought. MIT Press, Cambridge, pp 79–111

World Public Opinion Poll (2009) Public attitudes toward climate change: Findings from a multi-country poll. http://www.worldpublicopinion.org/pipa/pdf/dec09
